# Ultrasound to Assess the Temporomandibular Joint of Children With Juvenile Idiopathic Arthritis: A Systematic Review

**DOI:** 10.1155/ijod/2825133

**Published:** 2026-01-19

**Authors:** Jesse Chana, Kimia Baghaei, Nathalia Carolina Fernandes Fagundes, Abhilash Hareendranatan, Jacob L. Jaremko, Marinka Twilt, Fabiana T. Almeida

**Affiliations:** ^1^ Mike Petryk School of Dentistry, Faculty of Medicine and Dentistry, University of Alberta, Edmonton, Alberta, Canada, ualberta.ca; ^2^ Radiology and Diagnostic Imaging, Faculty of Medicine and Dentistry, University of Alberta, Edmonton, Alberta, Canada, ualberta.ca; ^3^ Cumming School of Medicine, Alberta Children’s Hospital, University of Calgary, Calgary, Alberta, Canada, ucalgary.ca

**Keywords:** juvenile idiopathic arthritis, temporomandibular joint, temporomandibular joint disorder, ultrasonography

## Abstract

**Objective:**

This systematic review assessed the diagnostic capability of ultrasound (US) to evaluate temporomandibular joint (TMJ) arthritis in children with Juvenile Idiopathic Arthritis (JIA), with MRI, CT, or CBCT as reference standards.

**Material and Methods:**

A search was conducted on electronic databases (Medline, Embase, Cochrane, Web of Science, and Scopus) and partial gray literature without restrictions of language and time. Eligibility criteria included diagnostic studies (randomized clinical trials, cohort studies, observational studies) evaluating the diagnostic potential of 2D or 3D US in assessing the TMJ (i.e., disc displacement, joint effusion, condylar changes) of children with JIA compared to CBCT/CT and/or MRI as the reference standard. The Quality Assessment Tool for Diagnostic Accuracy Studies‐2 (QUADAS‐2) was used to evaluate risk of bias.

**Results:**

After eligibility criteria were applied, eight studies were included. All studies were methodologically acceptable, presenting low applicability concerns, although none fulfilled all QUADAS‐2 criteria. Results from individual studies ranged from poor to excellent, with a sensitivity (SN) of B‐mode US ranging between 21% and 85% and a specificity (SP) between 36.4% and 89%, depending on the TMJ alterations assessed (condylar changes ‐ CC, joint effusions ‐ JE, synovial thickness, and lateral periarticular space ‐ LPAS). The certainty of the evidence for the diagnostic performance of US to detect TMJ involvement in JIA was graded low for SN and moderate for SP.

**Conclusion:**

This systematic review demonstrated that although MRI remains the reference standard imaging modality, US shows promising diagnostic capability in assessing the TMJ of children with JIA. It can provide valuable supplementary information to assist in diagnosis alongside clinical examination and guide the need for advanced diagnostic assessment.

## 1. Introduction

The temporomandibular joint (TMJ) can be the first joint or even the only one affected by Juvenile Idiopathic Arthritis (JIA), with more than 90% of children with JIA presenting with TMJ involvement during their disease [1, 2]. JIA is characterized by persistent inflammation of one or multiple joints with onset before the age of 16 [3]. It is one of the most common rheumatic diseases in children, with a prevalence of ~ 58–65 per 100,000 in developed countries [4].

TMJ arthritis has long been overlooked in the general care of JIA. However, over the past decade, there has been increasing awareness of the significant impact of TMJ arthritis on the overall health and well‐being of children with JIA [5]. These impacts include functional and esthetic consequences such as craniofacial deformities, limited mouth opening, and mandibular retrognathia and asymmetry [6, 7]. Signs and symptoms associated with TMJ arthritis, including pain, TMJ clicking and crepitus, and alteration in the growth of the mandible, can also significantly impact children’s quality of life. It’s known that JIA is a risk factor for the development of temporomandibular joint disorders (TMD), with TMJ alterations being more prevalent in children and adolescents with JIA [8].

TMJ arthritis in children is often associated with a delayed diagnosis due to a lack of obvious clinical symptoms and difficulties in examination [9]. Pain and swelling are not present in most JIA cases and, therefore, render clinical symptoms unreliable in the detection of TMJ involvement [9]. Damage from silent inflammation can progressively worsen damage as the condyle grows and can impair growth itself. Severe complications are often due to the lack of proper diagnosis and treatment delivered in a timely fashion, stressing the importance of early screening and diagnosis [8]. Imaging is considered the most sensitive and objective means of detecting TMJ arthritis. Diagnostic imaging can confirm or rule out suspected diseases and gather information unavailable during clinical examinations [10]. Magnetic resonance imaging (MRI) is the reference standard in detecting TMJ arthritis in children with JIA [11]. However, the downsides of MRI include high costs, which limit its availability to main centers and major cities, long wait times, and the need for sedation in younger children [12]. In addition, MRI has limited value in assessing TMJ bone changes. Cone beam computed tomography (CBCT) has been used to assess bone changes in TMJ; however, it cannot evaluate alterations in soft tissue. Specifically, it can not comment on signs of active inflammation in JIA patients. Not to mention, the considerable amount of radiation that can be harmful for children if used repeatedly limits its use [13].

Ultrasound (US) has been assessed in its ability to screen TMJ arthritis in JIA patients. US is non‐invasive, does not involve ionizing radiation, is low‐cost, and can be performed chair‐side in a portable manner [9]. This technology has significantly improved in recent years, and some studies have demonstrated its ability to detect TMJ inflammation, bony changes, and disease progression in JIA patients [7]. The ability of US to provide real‐time, dynamic evaluation makes it especially valuable for pediatric patients who may have difficulty undergoing traditional imaging procedures. However, published research findings remain inconsistent, with some studies suggesting that US is a potential diagnostic tool, whereas others state the opposite. A previous review found a moderate association between US and MRI TMJ findings in children with JIA [14]. Considering recent advances in US technology and its fast and increasing implementation in dentistry, this systematic review aimed to explore the current literature to assess the diagnostic capability of US for TMJ arthritis in children.

## 2. Materials and Methods

The systematic review was conducted following the recommendations of the “Preferred Reporting Items for a Systematic Review and Meta‐analysis of Diagnostic Test Accuracy Studies, PRISMA‐DTA Checklist” [15]. The review was registered in the Open Science Framework and can be found at the following address: https://osf.io/k6ut3/.

### 2.1. Study Design

A systematic review of human studies was undertaken to answer the following research question: “What is the diagnostic capability of US to assess TMJ arthritis (including disc displacement, joint effusions, and structural condylar changes) in children with JIA compared to MRI, CT, or CBCT as the reference standard?”

### 2.2. Eligibility Criteria

Diagnostic studies (randomized clinical trials, cohort studies, and observational studies) evaluating the diagnostic potential of 2D or 3D US imaging in assessing TMJ arthritis in children (under 18 years old) with JIA were included. The reference standard imaging was established with 3D imaging modalities (MRI, CT, or CBCT). Studies were included if JIA‐associated TMJ arthritis status was confirmed by a reference standard (MRI, CT, or CBCT), regardless of whether individual imaging features were directly compared between modalities. No language or time restrictions were set. Excluded criteria included: (1) reviews, letters, case reports, case series, personal opinions, book chapters, and conference abstracts; (2) studies without the reference standard comparison (MRI, CT, or CBCT); (3) incomplete data — studies without sensitivity (SN) and SP or ROC curve; and (4) studies assessing joints other than TMJ.

### 2.3. Information Sources and Search Strategy

Detailed individual search strategies for the following electronic databases were performed: Medline, Embase, Cochrane, Web of Science, and SCOPUS. A partial gray literature was accessed using Google Scholar by screening the abstracts for the first 100 results (filtered by “relevance”). All databases were initially searched on June 26^th^, 2024, and updated on July 24^th^, 2025. Appropriate truncation and word combinations were selected and adapted to each database search (Supporting Information 1: Appendix S1) using the expertise of a health sciences librarian.

### 2.4. Study Selection

A two‐phase selection of articles was conducted. In Phase 1, two authors (J.C. and F.T.A.) independently reviewed the references’ titles and abstracts. These authors selected articles that appeared to meet the inclusion criteria based on their titles and abstracts. In this phase, we used Covidence, a specific tool for the systematic review screening process, available at https://www.covidence.org/. In Phase 2, the same authors assessed the full text of all screened articles and excluded studies that did not meet the inclusion criteria. Disagreements between the two authors were solved by consensus. The final selections were always based on the full text of the publication.

### 2.5. Data Collection Process and Data Extraction

One author (J.C.) collected the required information from the included studies, and a second author (F.T.A.) cross‐checked all the collected data. For all included studies, the following information was extracted: study characteristics (author, year, country), study design and sample characteristics (population studied, age, sex), intervention characteristics (reference standard, index test, type, transducer frequency, target), and outcomes (main results and conclusion).

### 2.6. Risk of Bias Assessment

To assess the methodological quality and applicability of the included studies, the Quality Assessment Tool for Diagnostic Accuracy Studies‐2 (QUADAS‐2) was applied [16]. Two authors (J.C. and K.B.) independently evaluated the quality of each included study and scored each item as “yes,” “no,” or “unclear.” Disagreements were resolved by a third author (F.T.A.), an oral and maxillofacial radiologist with extensive experience in systematic reviews and 8 years of work with US technology.

### 2.7. Level of Evidence

The overall certainty of the evidence was assessed using the GRADEpro criteria (Grading of Recommendations Assessment, Development, and Evaluation—GRADEpro Guideline Development Tool [Software]. McMaster University and Evidence Prime, 2024) [17]. This tool assesses the level of evidence among outcomes of this systematic review regarding the risk of bias, inconsistency, indirectness, imprecision, and publication bias. The GRADE’s Summary of Findings (SoF) was adopted for data reporting.

### 2.8. Quantitative Analysis

Initially, we planned to add a quantitative meta‐analysis of the diagnostic test accuracy of US to detect TMJ involvement in JIA. However, this could not be meaningfully performed due to the high methodological heterogeneity observed across included studies. (i.e., evaluation of different TMD parameters for diagnosis among children with JIA and different US frequencies used).

## 3. Results

### 3.1. Study Selection

**Figure 1:**
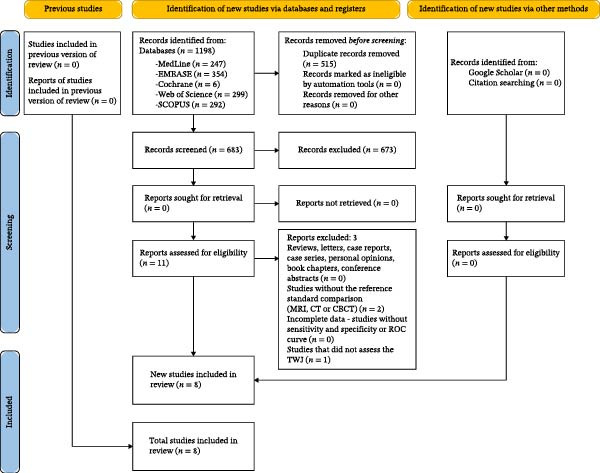
Prisma flow diagram.

The search identified 1198 articles. We removed 515 duplicates, and the titles and abstracts of 683 articles were screened. After assessing these articles in phase 1673 were excluded, and 11 were evaluated in full text for eligibility. Three studies were excluded based on the exclusion criteria (Supporting Information 2: Appendix S2). Finally, eight studies satisfied the inclusion criteria of this review and were included in the paper. The PRISMA flowchart (Figure 1) shows a flow diagram outlining the identification, screening, exclusion, and inclusion of studies.

### 3.2. Study Characteristics

The included studies were published from 2007 to 2025 and were all written in English. The studies were conducted in 6 countries (Germany, Norway, Switzerland, Italy, the USA, and Brazil). Study designs included four prospective studies, two pilot studies, one comparative study, and one observational diagnostic study. 312 JIA patients were assessed across the studies. The sample size ranged from 8 to 92 JIA children, all under 18 years of age. Five studies assessed joint effusion (JE). Two studies evaluated the lateral periarticular space (LPAS). One study assessed vascularization [18]. Other factors, such as condylar morphology, condylar erosions (CE), synovial thickening, subcondylar and condylar capsular width, and dynamic function, were also investigated. All included studies used MRI as the reference standard to confirm the presence of JIA‐associated TMJ involvement or to compare the alterations between the two imaging modalities. US frequencies ranged from 12 to 18 Megahertz (MHz). Zwir et al. [18] was the only study to investigate the diagnostic performance of power Doppler US. Studies scanned the TMJ extraorally in closed‐ and open‐mouth positions, except for Tonnie et al. [19] and Kirkhus et al. [20], who conducted only closed‐mouth scans. A summary of descriptive characteristics of studies in the included articles is given in Table 1.

**Table 1:** Summary of descriptive characteristics of included studies.

Study characteristics	Study design and sample characteristics		Intervention characteristics			Outcomes
Author, year Country	Population studied (age, sex)	Sample size and study design	Reference standard	Index test (type and frequency)	Target	Main results and conclusion
Assaf et al. [21], Germany	JIA patients Mean age 11.06 years 17 females and 3 males	(*n* = 20) Prospective study	MRI (strength of magnet NA)	High‐resolution US 12 MHz	CE, thickness of the condylar disc, synovial thickness, JE, and enlargement of the intra‐articular space	HR US detected 287 changes (35.9%) CE, 124 images (77.5%) Abnormal synovial thickness: 55 images (34.4%) Higher thickness of condylar disc: 48 images (30%) Irregularities of bony surface: 40 images (25%) JE, 20 images (12.5%) Conclusion: HR US could be a diagnostic tool for JIA, even if all parts of the TMJ are not visible for US (especially for condylar involvement)
Farronato et al. [7], Italy	JIA patients Mean age 15.4 years 39 females and 7 males	(*n* = 46) Prospective study	MRI (strength of magnet NA)	High‐resolution US (12–18 MHz); linear probe	Condylar morphology, disc position, soft tissue changes (joint capsule thickness), and dynamic function. JE	Diagnostic Accuracy‐US vs. MRI in 30 patients SN, 85%; SP, 80% PPV, 88%; NPV, 75% HR US detected TMJ abnormalities in 39 patients with JIA (84.8%) Conclusion: US is a valuable adjunctive tool for evaluating TMJ involvement in children with JIA
Kirkhus et al. [20], Norway	JIA patients Mean age 12.4 years 42 females and 13 males	(*n* = 55) Retrospective study	Contrast‐ enhanced MRI (1.5 T)	High‐resolution US (12–18 MHz); linear probe	Subcondylar and condylar capsular width	TMJ Capsular Width SN, 72%; SP, 70% Conclusion: US may be a valuable diagnostic tool in the initial assessment of TMJ inflammation since it moderately correlates with MRI for TMJ inflammation
Müller et al. [22], Switzerland	JIA patients 16 females and 14 males Age at examination, median (range), years 9.8 (2.8–16.9)	(*n* = 30) Pilot study	Contrast‐ enhanced MRI (1.5 T)	US 12 MHz; linear probe	JE and/or increased contrast enhancement of synovium or bone = active arthritis (in MRI)	SN, 23%; SP, 89% Conclusion: US can only detect the most severely affected TMJs Therefore, US is not recommended as a screening method for early TMJ involvement in patients with JIA
Tonni et al. [23], Italy	JIA patients 8 JIA children (7 girls 1 boy) mean age: 11.6 ± 3.5 years 7 healthy children (4 girls and 3 boys) mean age 9.3 ± 1.2 years	JIA (*n* = 8) Control group (*n* = 7) Pilot study	Contrast‐ enhanced MRI (1.5 T)	US 15 MHz; linear probe	LPAS and JE	LPAS values were higher in the JIA group (*p* < 0,001). US can detect differences in TMJ features between JIA patients and healthy individuals Conclusion: It may serve as a potential follow‐up tool for assessing TMJ involvement in patients with JIA
Tonni et al. [19], Italy	JIA patients (26 females, 3 males). Mean age: 13 ± 2.8 years Healthy control children (24 girls and 4 boys). Mean age: 12.6 ± 2.5 years	JIA (*n* = 29) Control healthy group (*n* = 28) Comparative study	MRI (1.5 T)	US 15 MHz	LPAS width	A positive significant correlation between MRI and US for LPAS width measurement was found. US could be used as a supplementary imaging method for assessing TMJ disease in JIA patients
Weiss et al. [24], USA	Children with new‐onset JIA 25 females, 7 males median age 8.6 years (range 1.5–17.2 years)	JIA (*n* = 32) Prospective study	MRI (1.5 T)	US 12.5 MHz; linear probe	Chronic TMJ arthritis (condylar changes/erosions) Acute TMJ arthritis (effusions, synovial thickening)	US with lower sensitivity for detecting TMJ arthritis compared to MRI 23% agreement between MRI and US for acute TMJ arthritis (kappa 0.00) 50% agreement between MRI and US for chronic TMJ changes (kappa 0.12) Conclusions: MRI and US results are not well correlated. MRI is preferable for the detection of TMJ disease in new‐onset JIA
Zwir et al. [18], Brazil	JIA patients 63 females, 29 males Mean age: 12.7 years	JIA (*n* = 92) Observational diagnostic study	Contrast‐ enhanced MRI (1.5 T)	US Gray‐scale (13 MHz) and Power Doppler (6.7 MHz)	Synovial vascularity, synovial inflammation	SN, 0%; SP, 36.4% Conclusion: Power Doppler US cannot replace MRI for the detection of TMJ inflammation in patients with JIA

Abbreviations: CE, condylar erosions; HR, high‐resolution; JE, joint effusion; JIA, juvenile idiopathic arthritis; LPAS, lateral periarticular space; MRI, magnetic resonance imaging; NA, not available; SN, sensitivity; SP, specificity; TMJ, temporomandibular joint; US, ultrasound.

### 3.3. Risk of Bias (RoB) and Applicability

**Figure 2:**
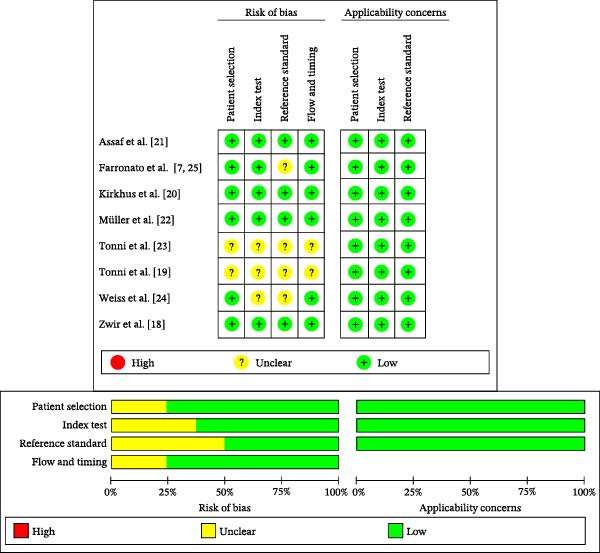
QUADAS risk of bias assessment.

None of the studies fulfilled all the methodological quality criteria. The RoB for patient selection was scored as “low” for all except for two studies [19, 23], which were scored as “unclear.” The RoB for the index test domain was scored as “low” for five studies and “unclear” for three. Likewise, the RoB for the reference standard domain was scored as “low” for four studies and “unclear” for four. The RoB for the flow and timing domain scored “low” for six studies and “unclear” for two. All studies presented low applicability concerns in the patient selection, index test, and reference standard domains (Figure 2). Supporting Information 3: Appendix S3 details the QUADAS‐2 assessment criteria for each included study.

### 3.4. Results of Individual Studies

The definition and criteria for assessing each TMJ finding in US varied considerably across studies (Table 2). Joint effusion was inconsistently defined, ranging from hypoechoic areas to unspecified fluid collections. Condylar erosion was described as cortical irregularity or loss, while LPAS and synovial inflammation were evaluated in only a few studies with different thresholds.

**Table 2:** TMJ findings assessed by US with their definitions.

Study (author/year)	Joint effusion (JE)	Condylar changes	LPAS (lateral periarticular space)	Synovial thickness/inflammation	Disc
Assaf et al. [21]	Sonographicvisible fluid accumulation	Condylar surface irregularity (yes/no) Condylar erosion (CE): hyperechoic reflection width	NA	Synovial thickening and fluid collection	Thickness of the condylar disc
Farronato et al. [7]	Loss of parallelism between the articular surfaces	Condylar morphology: regular vs. irregular shape; irregularity defined as alteration in smooth contour; flattening defined as reduced convexity; prominence defined as protruded contour vs. expected morphology CE, defined as cortical discontinuity or hypoechoic defects along the condylar surface	NA	Thickened joint capsule (>0.1 cm)	Disc position (normal vs. displaced on opening/closing); periarticular soft tissue swelling; real‐time condylar movement (range of motion, excursion, lateral movements, protrusion)
Kirkhus et al. [20]	NA	Capsular shape (not included in results)	NA	Capsular width as an indirect measurement of synovitis	NA
Müller et al. [22]	Not clearly defined (anechoic area)	Surface irregularity, flattening CE, absence of cortical lining	NA	Thickened joint capsule (>2 mm)	NA
Tonni et al. [19]	Hypoechoic area within the articular space	NA	Synovial joint space: hypoechoic gap between the lateral condylar cortex and the joint capsule	NA	NA
Tonni et al. [19]	NA	NA	Synovial joint space: hypoechoic gap between the lateral condylar cortex and the joint capsule	NA	NA
Zwir et al. [18]	NA	NA	—	Positive power Doppler signal	NA
Weiss et al. [24]	Fluid collection: (not clearly defined)	Condylar changes CE, not clearly defined	NA	NA	NA

Abbreviation: NA, not assessed.

Most of the studies (*n* = 7) used B‐mode US with frequencies ranging between 12 and 18 MHz. US diagnostic performance in these studies varied, with SN ranging from 21% to 85% and SP between 36.4% and 89%, depending on the TMJ finding assessed. In a prospective study in Germany, Assaf et al. [21] examined 20 children with a mean age of 11.1 years using high‐resolution ultrasonography (12 MHz, linear‐array transducer) to evaluate the TMJ. MRI served as the reference standard to confirm JIA‐associated TMJ arthritis status, and US findings were interpreted accordingly to assess its ability to detect affected joints. The assessment examined five domains: condylar erosion (CE), the thickness of the condylar disc, synovial thickness, joint effusion (JE), and the enlargement of the intra‐articular space. The scans were performed with the patient lying on one side, in two positions: mouth closed (parallel to the Frankfurt horizontal plane) and maximum opening (60–70° to the Frankfurt horizontal plane). Upon examination, 287 (35.9%) changes were detected, including the following: CE on 124 (77.5%) images, increased thickness of the condylar disc in 48 images (30%), abnormal synovial thickness in 55 (34.4%) of images, JE in 20 images (12.5%), and irregularities of the bony surface on 40 images (25%). These results demonstrated the diagnostic potential of US in detecting TMJ changes.

Zwir et al. [17] explored the diagnostic potential of Doppler US to assess TMJ synovial inflammation in 92 patients with a mean age of 12.7 years. The scans were conducted with a 6.7 MHz frequency (multi‐frequency, linear‐array transducer) using Doppler synovial vascularity and synovial enhancement as determinants of the synovial inflammation. The scans were done along the long axis of the mandibular ramus in both open and closed‐mouth positions. MRI reported inflammation in 119 joints (64.7%), while power Doppler US reported none, resulting in 0% SN, 36.4% SP, 0% positive predictive value (PPV), and a 100% negative predictive value (NPV) for active inflammation. The study showed that power Doppler is not a useful diagnostic tool for TMJ synovial inflammation.

In a pilot study, Müller et al. [22] assessed 30 patients with a median age of 9.8 years at examination. The following were examined: JE and/or increased contrast enhancement of synovium or bone. The index test used was a 12 MHz US linear‐array transducer. TMJ was assessed dynamically during mouth opening. Transverse and coronal static US images of the mandibular condyles were obtained in both the closed and maximally open‐mouth positions. The reference standard used was contrast‐enhanced MRI and clinical examination (rheumatological and orthodontic examinations). They found that US with pathological findings was statistically correlated with TMJ pathology (chi‐square *p* = 0.002) and active TMJ arthritis on MRI (chi‐square *p* = 0.008 for all joints and *p* = 0.047 for patients). TMJ with severe enhancement was correctly diagnosed as pathological 7/8 (88%). However, TMJ with mild enhancement was only correctly diagnosed 7/20 (35%) of the time (chi‐square *p* = 0.003 for the difference between the groups). They noticed that the US could only detect the most severely affected TMJs with an SN of 23% and an SP of 89%.

Weiss et al. [22] conducted a prospective study in the USA examining 32 children with new‐onset JIA, with a median age of 8.6 years. The US images were obtained in the coronal and axial planes, with the patient in either an open‐mouth or closed‐mouth position. They assessed TMJ arthritis, defined by alterations such as condylar changes/erosions, effusions, synovial thickening, and fluid collection in either joint. The index test used was a 12.5 MHz linear‐array transducer US, intending to compare US ability to detect acute and chronic changes of TMJ arthritis. They found chronic arthritis in 22 (69%) patients by MRI and 9 (28%) patients by US; the two modalities had 50% agreement and kappa = 0.12. However, US could not detect acute TMJ arthritis at all (kappa value 0,) while MRI detected it in 24 (75%) patients, reporting a 23% agreement.

Additionally, in a retrospective study in Norway, Kirkhus et al. [20] examined 55 children (mean age 12.4 years). The index test was a high‐frequency 12–18 MHz US with a linear‐array transducer. A longitudinal ultrasonography image of the TMJ in a closed‐mouth position was obtained. They assessed subcondylar and condylar capsular width and US’s ability to detect synovitis. Results found a moderate correlation between US‐assessed capsular width and MRI‐assessed synovitis both at the condylar and subcondylar levels [Spearman’s rho (*r*): 0.483; *p*, 0.001 and 0.347; *p*, 0.001, respectively]. A receiving operating characteristic (ROC) curve demonstrated the best discriminatory ability at the subcondylar level with an area under the curve of 0.77 (95% confidence interval 0.69–0.85) and a cut‐off value of 1.2 mm (SN 72%, SP 70%) for the capsular width. This demonstrates that the US is preferred at a subcondylar level and may serve as a valuable diagnostic in the initial assessment of TMJ inflammation.

Tonni et al. [23] conducted a pilot study in Italy to assess eight patients with the mean age of 11.6 years. The index test was a 15 MHz US, assessing LPAS and JE in closed and open‐mouth positions. In a comparative study based in Italy, Tonni et al. [19] looked at 29 JIA children with a mean age of 13 ± 2.8 years. The index test used was a 15 MHz US to assess LPAS width only in the closed‐mouth position. MRI images resulted in a Spearman Rank Correlation that was moderately positive with a ρ of 0.623 and a *p*  < 0.05. However, the control and JIA groups did not show any signs of JE [20]. Similarly, Tonni et al. [19] found that in the JIA group, the Spearman correlation coefficient showed a positive, significant correlation (*r* = 0.403, *p* = 0.002). Additionally, a good level of agreement was found between measurements of LPAS for both US and MRI.

A recent study from Farronato et al. [25] evaluated 46 patients with a mean age of 15.4 years using high‐frequency US (12–18 MHz) to detect TMJ alterations. The examination included assessment of condylar morphology, disc position, soft tissue changes, and dynamic function. The US detected condylar irregularities in 56.5%, flattening in 34.8%, prominence in 28.3%, erosive changes in 8.7%, restricted condylar excursion in 39.1%, soft tissue abnormalities in 21.7%, and joint effusion in 6.5% of patients. Comparison with MRI in 30 patients showed a SN of 85%, SP of 80%, positive predictive value of 88%, and negative predictive value of 75% for detecting TMJ pathology. The study demonstrated that US has good concordance with MRI and may serve as a non‐invasive tool for routine monitoring of TMJ in JIA.

### 3.5. Certainty of Evidence

The certainty of the evidence collected from six of the included studies for the performance of US to detect TMJ involvement in JIA was graded as low regarding SN and moderate regarding SP. These results were mainly influenced by the unclear risk of bias for some categories in most studies and the significant variability of sensitivity results (i.e., inconsistency) across studies (Supporting Information 4: Appendix S4). Assaf et al. [22] and Farronato et al. [25] were not included in the certainty of evidence assessment of true negatives and false positives due to the lack of participants without JIA in the sample.

## 4. Discussion

This systematic review showed that US diagnostic performance in evaluating TMJ arthritis in children with JIA varies from poor to very good (SN: 21% to 85% and SP between 36.4% and 89%), depending on the TMJ alteration assessed. The wide variability in diagnostic performance observed across studies may be due to methodological heterogeneity, partly attributed to differences in US equipment, scanning protocols, and the various definitions applied for each TMJ finding investigated. Key diagnostic features, such as JE and CE, lacked standardized definitions. For example, CE definitions ranged from loss of hyperechoic contour to surface irregularity in the image.

Five of the eight included studies concluded that US can be used as a diagnostic tool in TMJ JIA [19–21, 23, 25]. These studies indicated that US can be used as an adjunct tool to MRI for screening and monitoring TMJ changes, particularly valuable for assessing synovial width, CE, and LPAS. This is promising, given that the US is significantly cheaper than MRI and that MRI often requires sedation in young children. Other US characteristics, such as portability, accessibility, non‐invasiveness, and being radiation‐free, facilitate safer and more accessible disease screening and monitoring.

Conversely, three studies demonstrated opposite results, suggesting that US should not be used as a screening tool to assess TMJ changes [18, 22, 24]. Müller et al. [22] and Weiss et al. [24] found that US and MRI values do not correlate well, with US detecting only severely affected TMJs or chronic disease. As mentioned by one of them [22], with the improvement of this technology and its increased use, the experience with TMJ US is expected to grow. Notably, these studies were conducted more than 15 years ago. With the rapid evolution of the US in recent years and increasing exposure of oral radiologists and dental clinicians to this technology, this result might not represent today’s SN and value. Additionally, both studies used US machines operating at 12 MHz, a lower frequency than those that reported superior results.

It is well‐known that US imaging is machine‐dependent and operator‐dependent. Therefore, the type of transducer and the frequency are essential when translating the results. Older studies using lower‐frequency transducers [14] tended to report lower sensitivity, while Kirkhus et al. [20] using a 12–18 MHz range, showed comparatively better performance. One possible technical explanation is that lower frequencies could be more effective for detecting inflammatory changes, whereas higher frequencies might better capture bony alterations. However, most included studies used transducers within the 12–18 MHz range [18, 19, 21, 23, 25], limiting our ability to determine whether specific frequency ranges consistently improve diagnostic accuracy. Standardized scanning protocols incorporating recent US technologies should be optimized in future studies.

The poor US SN to assess active TMJ inflammation in some studies is somewhat surprising, since in other superficial joints, such as the hands, US is well recognized to detect active inflammation (synovitis/effusion) usefully [26]. It may be that active arthritis progresses more slowly at this joint compared to other joints with little synovitis to detect. Although the US holds greater diagnostic potential than clinical examination [27], US imaging alone may not have sufficient sensitivity to assess TMJ in JIA. Therefore, combining the US with clinical evaluations and patient symptoms could improve its diagnostic performance, helping identify patients requiring further MRI. While the US is not a replacement for MRI, it may serve as a valuable tool in conjunction with other clinical markers to guide the need for further diagnostic assessment, helping with the disease triage. Nevertheless, the US performed better in assessing parameters like synovial width, CE, and LPAS; therefore, developing a standardized US protocol to evaluate each alteration is needed.

Zwir et al. [18] explored the potential of power Doppler and found it not to be a valid diagnostic tool to assess TMJ synovial inflammation. This may be due to technical features like motion artifacts during TMJ assessment and/or relatively low‐grade hyperemia in TMJ in JIA, which might not dramatically increase blood flow in periarticular structures. By contrast, power Doppler has proven valuable for synovitis imaging in other joints (e.g., small joints of the hands, wrists, and knees) in children with JIA. For instance, synovial hyperemia (active inflammation) has been detected as a positive power Doppler signal in JIA studies assessing joints like knees and ankles [28, 29]. Therefore, caution is required when generalizing the results of this study. Although power Doppler seems to bring minimal contribution to the TMJ assessment, this is a result of one study, and future studies might want to explore its potential.

The GRADE assessment showed low certainty of evidence for SN and moderate certainty for SP across the included studies. This suggests that current evidence on US SN is limited and subject to change with future research. Clinically, low SN means that US may miss a significant number of TMJ arthritis cases, limiting its reliability as a standalone screening tool. In contrast, moderate SP implies that while false positives may occur, a positive US finding has some diagnostic value. Together, these findings support the role of US as an adjunct tool, rather than a replacement for MRI, and highlight the need for better standardization and larger studies to improve confidence in US‐based diagnosis.

A few limitations should be discussed. First, there is an unclear risk of bias in the index and reference tests domains across the included studies. JIA can impact TMJ bone development, leading to changes such as condylar erosions, flattening, and asymmetry, which are best visualized with CBCT. Unlike MRI, which cannot capture cortical bone detail, CBCT offers high‐resolution imaging of osseous structures and is thus indispensable for assessing long‐term joint damage. None of the included studies used CBCT as a reference standard. Future studies should directly compare US and CBCT, particularly to evaluate whether US can detect superficial cortical irregularities and determine its potential as a complementary tool for early diagnosis and monitoring of TMJ bone changes in JIA. Second, the included studies exhibited methodological heterogeneity, particularly in the parameters assessed when evaluating the TMJ, which prevented a formal quantitative analysis. In addition, variability was observed in how the reference standard was applied. Some studies used the reference standard to confirm overall TMJ arthritis status, whereas others provided feature‐level (e.g., disk displacement, effusion, condylar alterations) comparisons with US. Another limitation relates to the variability in diagnostic performance threshold reported across the studies. As an example, while Tonni et al. [23] presented moderate‐to‐strong correlations between US and MRI findings (*ρ* = 0.623), others, such as Kirkhus et al. [20] reported lower coefficients (*ρ* = 0.347–0.483) and considered it moderate. This inconsistency in outcome reporting limits the ability to directly compare diagnostic accuracy across studies. A standardized protocol for TMJ US image acquisition and interpretation is still lacking. Future studies should make efforts to establish a protocol including what should be assessed and assessment criteria and establish normal parameters for evaluating the TMJ in children, ensuring assessment consistency and accuracy. Finally, methodological issues and heterogeneity may impact the generalizability of the results. The number of studies addressing the topic and the number of patients included may also affect the assessment of US diagnostic performance for this purpose.

US is highly desirable for assessing TMJ arthritis in children as a quick, cost‐effective, non‐invasive, and portable initial screening test. There is an opportunity to evaluate whether using optimized scan protocols with modern portable US incorporated into AI image analysis could improve the clinical utility of TMJ US. It is important to note that all studies used a conventional full‐sized “cart” US. Future studies investigating the potential of handheld pocket US to assess the TMJ are encouraged, as it is much more practical for use at clinical points of care where access to MRI is limited. If problems with low test sensitivity can be successfully addressed, the US could be a valuable tool to screen for TMJ pathologies in children with JIA. Ideally, this would minimize costly MRI and CT scans with ionizing radiation in clinical care pathways.

## 5. Conclusion

This systematic review demonstrated that although MRI remains the standard imaging modality for assessing TMJ in children with JIA, the US shows promising diagnostic capability and may be a potential diagnostic aid in screening and monitoring TMJ if following a specific protocol and standard observations. It can provide valuable supporting information to assist in diagnosis alongside clinical examination and guide the need for advanced diagnostic assessment.

## Funding

This research was supported by the Women and Children’s Health Research Institute (Grant ID3915).

## Disclosure

All authors have reviewed and approved the final version of the manuscript and agree with its submission to the journal.

## Conflicts of Interest

The authors declare no conflicts of interest.

## Supporting Information

Additional supporting information can be found online in the Supporting Information section.

## Supporting information


**Supporting Information 1** Appendix S1: Search strategies with appropriate key words and MeSH terms.

**Supporting Information 2** Appendix S2: Excluded articles and reasons for exclusion.

**Supporting Information 3** Appendix S3: Risk of Bias in Individual Studies. QUADAS‐2 criteria fulfilled.

**Supporting Information 4** Appendix S4: Certainty of evidence across included studies.

## Data Availability

Data sharing is not applicable to this article, as no datasets were generated or analyzed during the current study.
