# Supplementary material for: Ultrasound to Assess the Temporomandibular Joint of Children With Juvenile Idiopathic Arthritis: A Systematic Review
Source: Int J Dent. 2026 Jan 19;2026:2825133. doi: 10.1155/ijod/2825133 (PMC12815698; doi:10.1155/ijod/2825133)
Supplement: Supplementary file 1 — Supporting Information 1 Appendix S1: Search strategies with appropriate key words and MeSH terms. [file IJOD-2026-2825133-s001.docx]

**Appendix 1.** Search strategies with appropriate key words and MeSH terms.

| Database | Search strategy  (Search updated July 24th, 2025)  n=1,198 |
| --- | --- |
| Cochrane  Results: 6 | ID Search  #1 juvenile* NEAR/2 arthritis  #2 MeSH descriptor: [Arthritis, Juvenile] explode all trees  #3 #1 OR #2  #4 ultrasound*  #5 MeSH descriptor: [Ultrasonography] explode all trees  #6 ultrasonograph*  #7 sonograph*  #8 magnetic resonance imag*  #9 Diagnostic Imaging*  #10 MeSH descriptor: [Diagnostic Imaging] explode all trees  #11 3D  #12 CT  #13 Computerized tomography  #14 {OR #4-#13}  #15 temporomandibular  #16 MeSH descriptor: [Temporomandibular Joint Disorders] explode all trees  #17 (condylar NEAR/2 (effusion* or change* or degenerat* or alteration* or resorption* or erosion*))  #18 disc displacement*  #19 {OR #15-#18}  #20 #3 AND #14 AND #19 |
| Embase  Results: 354 | 1. (juvenile* adj3 arthritis).mp. or exp juvenile arthritis/2. ultrasound.mp.3. exp Ultrasonography/ or ultrasonograph*.mp.4. 3D.mp.5. MRI.mp.6. magnetic resonance imag*.mp.7. CT.mp.8. exp x-ray computed tomography/9. exp Diagnostic Imaging/10. 2 or 3 or 4 or 5 or 6 or 7 or 8 or 911. temporomandibular.mp.12. exp temporomandibular joint disorder/ or exp mandible condyle/ or exp temporomandibular joint/13. disc displacement*.mp.14. 11 or 12 or 1315. 1 and 10 and 14 |
| Medline  Results: 247 results | 1. (juvenile* adj3 arthritis).mp.2. exp Arthritis, Juvenile/3. 1 or 24. ultrasound*.mp.5. exp Ultrasonography/ or ultrasonograph*.mp.6. sonograph*.mp.7. 3D.mp.8. exp Magnetic Resonance Imaging/ or MRI.mp.9. exp Magnetic Resonance Imaging/ or magnetic resonance imag*.mp.10. exp Tomography, X-Ray Computed/ or CT.mp.11. computerized tomography.mp. or exp Tomography, X-Ray Computed/12. exp Diagnostic Imaging/13. 4 or 5 or 6 or 7 or 8 or 9 or 10 or 11 or 1214. temporomandibular.mp.15. (condylar adj2 (effusion* or change* or degenerat* or alteration* or resorption* or erosion*)).mp.16. disc displacement*.mp.17. 14 or 15 or 1618. 3 and 13 and 17 |
| Web Of Science (All Databases)  Results: 299 | TS=(juvenile* NEAR/3 arthritis) AND TS=(ultrasound* OR ultrasonograph* OR sonograph* OR 3D OR MRI ) AND TS=(temporomandibular OR (condylar NEAR/2 (effusion* or change* or degenerat* or alteration* or resorption* or erosion*)) OR "disc displacement*" OR "joint effusion") |
| Scopus  Results: 292 | TITLE-ABS-KEY ( juvenile* W/3 arthritis ) AND TITLE-ABS-KEY ( ultrasound* OR ultrasonograph* OR sonograph* OR 3d OR mri OR ct ) AND TITLE-ABS-KEY ( temporomandibular OR ( condylar W/2 ( effusion* OR change* OR degenerat* OR alteration* OR resorption* OR erosion* ) ) OR "disc displacement*" OR "joint effusion" ) |
| Google Scholar | Juvenile Idiopathic Arthritis and Temporomandibular joint and Ultrasound  Sort by relevance the first 100. Without citations and patents |

Web of Science Core Collection

MEDLINE®

Current Contents Connect

183SIS Citation Index

BIOSIS Previews CABI: CAB Abstracts®

SciELO Citation Index ProQuest™ Dissertations & Theses Citation Index
