# Supplementary material for: Ultrasound to Assess the Temporomandibular Joint of Children With Juvenile Idiopathic Arthritis: A Systematic Review
Source: Int J Dent. 2026 Jan 19;2026:2825133. doi: 10.1155/ijod/2825133 (PMC12815698; doi:10.1155/ijod/2825133)
Supplement: Supplementary file 2 — Supporting Information 2 Appendix S2: Excluded articles and reasons for exclusion. [file IJOD-2026-2825133-s002.docx]

**Appendix S2.** Excluded articles and reasons for exclusion (n=3)

| **Author, Year** | **Reason for exclusion** |
| --- | --- |
| De Lucia et al., 2018 | (2) |
| Laurell et al., 2012 | (4) |
| Dev, Verma, and Singh, 2019 | (2) |

1. Reviews, letters, case reports, case series, personal opinions, book chapters, conference abstracts (n=0);
2. Studies without the reference standard comparison (MRI, CT or CBCT) (n=2);
3. Incomplete data - studies without sensitivity and specificity or ROC curve (n=0);
4. Studies assessing joints other than TMJ (n=1)

**References**

1. De Lucia O, Ravagnani V, Pregnolato F, et al. Baseline ultrasound examination as possible predictor of relapse in patients affected by juvenile idiopathic arthritis (JIA). *Ann Rheum Dis.* 2018;77(10):1426-1431. doi:10.1136/annrheumdis-2017-211696
2. Laurell L, Court-Payen M, Nielsen S, et al. Comparison of ultrasonography with Doppler and MRI for assessment of disease activity in juvenile idiopathic arthritis: a pilot study. *Pediatr Rheumatol.* 2012;10:23. doi:10.1186/1546-0096-10-23
3. Dev S, Verma A, Singh A. Musculoskeletal ultrasonography in detecting disease activity in patients of juvenile idiopathic arthritis: a cross-sectional study. *Indian J Rheumatol.* 2019;14(2):104-108. doi:10.4103/injr.injr_38_19
