# Supplementary material for: Ultrasound to Assess the Temporomandibular Joint of Children With Juvenile Idiopathic Arthritis: A Systematic Review
Source: Int J Dent. 2026 Jan 19;2026:2825133. doi: 10.1155/ijod/2825133 (PMC12815698; doi:10.1155/ijod/2825133)
Supplement: Supplementary file 3 — Supporting Information 3 Appendix S3: Risk of Bias in Individual Studies. QUADAS‐2 criteria fulfilled. [file IJOD-2026-2825133-s003.docx]

Appendix 3*.* Risk of Bias in Individual Studies. QUADAS 2 criteria fulfilled**.**

|  | Item | Assafa et al, 2013 | Farronato et al, 2025 | Kirkhus et al, 2016 | Muller et al, 2009 | Tonni et al, 2021 | Tonni et al, 2023 | Weiss et al, 2008 | Zwir et al, 2020 |
| --- | --- | --- | --- | --- | --- | --- | --- | --- | --- |
| Domain 1:  Patient  Selection | Was a consecutive or random sample of patients enrolled? | Y | Y | Y | Y | Y | Y | Y | Y |
|  | Was a case-control design avoided? | Y | Y | Y | Y | N | N | Y | Y |
|  | Did the study avoid inappropriate exclusions? | Y | Y | U | Y | U | U | Y | U |
|  | **Could the selection of patients have introduced bias?** | L | L | L | L | U | U | L | L |
|  | **Concerns regarding applicability:** Is there concern that the included patients do not match the review question? | L | L | L | L | L | L | L | L |
| Domain 2:  Index Test | Were the index test results interpreted without knowledge of the results of the reference standard? | Y | Y | Y | Y | U | U | U | Y |
|  | If a threshold was used, was it pre-specified? | U | U | U | U | U | U | U | U |
|  | **Could the conduct or interpretation of the index test have introduced bias?** | L | L | L | L | U | U | U | L |
|  | **Concerns regarding applicability:** Is there concern that the index test, its conduct, or interpretation differ from the review question? | L | L | L | L | L | L | L | L |
| Domain 3:  Reference Standard | Is the reference standard likely to correctly classify the target condition? | Y | Y | Y | Y | Y | Y | Y | Y |
|  | Were the reference standard results interpreted without knowledge of the results of the index test? | Y | U | Y | Y | U | U | U | Y |
|  | **Could the reference standard, its conduct, or its interpretation have introduced bias?** | L | U | L | L | U | U | U | L |
|  | **Concerns regarding applicability:** Is there concern that the target condition as defined by the reference standard does not match the review question? | L | L | L | L | L | L | L | L |
| Domain 4:  Flow and Timing | Was there an appropriate interval between index test(s) and reference standard? | Y | Y | Y | Y | Y | Y | Y | Y |
|  | Did patients receive the same reference standard? | Y | Y | Y | Y | N | N | Y | Y |
|  | Were all patients included in the analysis? | Y | Y | Y | Y | Y | Y | Y | Y |
|  | **Could the patient flow have introduced bias?** | L | L | L | L | U | U | L | L |

Yes (Y), no (N), unclear (U). Risk: LOW (L) /HIGH (H) /UNCLEAR (U)
