# Supplementary material for: Ultrasound to Assess the Temporomandibular Joint of Children With Juvenile Idiopathic Arthritis: A Systematic Review
Source: Int J Dent. 2026 Jan 19;2026:2825133. doi: 10.1155/ijod/2825133 (PMC12815698; doi:10.1155/ijod/2825133)
Supplement: Supplementary file 4 — Supporting Information 4 Appendix S4: Certainty of evidence across included studies. [file IJOD-2026-2825133-s004.docx]

Appendix 4: Certainty of evidence across included studies.

| **Outcome** | **№ of studies (№ of patients)** | **Study design** | **Factors that may decrease the certainty of evidence** | | | | |  |
| --- | --- | --- | --- | --- | --- | --- | --- | --- |
|  |  |  | **Risk of bias** | **Indirectness** | **Inconsistency** | **Imprecision** | **Publication bias** |  |
| **True positives** (patients with TMJ involvement) | 7studies 242 patients | cross-sectional (cohort type accuracy study) | serious^a^ | not serious | not serious | not serious | none | ⨁⨁⨁◯ Moderate^a^ |
| **False negatives** (patients incorrectly classified as not having TMJ involvement) |  |  |  |  |  |  |  |  |
| **True negatives** (patients without TMJ involvement) | 5studies 110 patients | cross-sectional (cohort type accuracy study) | serious^a^ | not serious | serious^b^ | not serious | none | ⨁⨁◯◯ Low^a,b^ |
| **False positives** (patients incorrectly classified as having TMJ involvement) |  |  |  |  |  |  |  |  |

#### **Explanations**

a. Unclear risk of Bias was identified for most of the studies in Index test and Reference test domain.

b. A large variability of results across studies was identified
